# Chemical nature of alkaline polyphosphate boundary film at heated rubbing surfaces

**DOI:** 10.1038/srep26008

**Published:** 2016-05-16

**Authors:** Shanhong Wan, A. Kiet Tieu, Qiang Zhu, Hongtao Zhu, Shaogang Cui, David R. G. Mitchell, Charlie Kong, Bruce Cowie, John A. Denman, Rong Liu

**Affiliations:** 1 Faculty of Engineering and Information Sciences, University of Wollongong, Wollongong, NSW 2522, Australia; 2 Electron Microscopy Centre, University of Wollongong, Wollongong, NSW 2522, Australia; 3 Electron Microscope Unit, The University of New South Wales, Sydney, NSW 2052, Australia; 4 Australian Synchrotron, 800 Blackburn Road, Clayton, Victoria 3168, Australia; 5 Future Industries Institute, University of South Australia, Mawson Lakes SA 5095, Australia; 6 Centre for Microscopy, Characterisation and Analysis, The University of Western Australia, Crawley, Australia

## Abstract

Alkaline polyphosphate has been demonstrated to be able to reduce significant wear and friction of sliding interfaces under heavy loads (>1 GPa) and elevated temperature (800 °C and above) conditions, e.g. hot metal manufacturing. The chemical composition and fine structure of polyphosphate lubricating film is not well understood as well as the role of alkaline elements within the reaction film at hot rubbing surface. This work makes use of the coupling surface analytical techniques on the alkaline polyphosphate tribofilm, XANES, TOF-SIMS and FIB/TEM. The data show the composition in gradient distribution and trilaminar structure of tribofilm: a shorter chain phosphate overlying a long chain polyphosphate that adheres onto oxide steel base through a short chain phosphate. The chemical hardness model well explains the anti-abrasive mechanism of alkaline polyphosphate at elevated temperatures and also predicts a depolymerisation and simultaneous cross-linking of the polyphosphate glass. The role of alkaline elements in the lubrication mechanism is especially explained. This work firstly serves as a basis for a detailed study of alkaline polyphosphate tribofilm at temperature over 600 °C.

Increasing demand on improved performance, service life and reliability is required in various machinery components that operate especially at elevated temperatures in internal combustion engines, bearings in aerospace propulsion systems, cutting tools, and metalworking processes^1^. Especially for manufacturing industry, more advanced metal manufacturing often requires increased tool forces and operation temperatures which lead to more heat and friction, no lubricant or ineffective lubrication will produce a higher friction, excessive wear, and severe oxidation as the rubbing metal surface is exposed to oxygen and water environment at elevated temperatures^2^. For today’s challenging manufacturing processes, the effective cooling and lubrication provided by the right lubricant is required to provide an extension of machinery components service life by orders of magnitude, resulting in considerable energy and material savings. Currently, the most successful anti-wear additive is a class of molecules called zinc dialkyldithiophosphate (ZDDP) that has been widely applied in engine industry. It can form an antiwear polyphosphate tribofilm on the contact area as a result of the interactions between the metallic surface and ZDDP^3^. This tribofilm performs as sacrificially adaptive film and, is constantly produced during frictional process, reduced friction and wear ultimately results^4^. That is, the growth of tribofilm is self-controllable, and this film embodies a supporting effect in response to different shear stressed conditions^5^. This has inspired us to develop new polyphosphate additives without heavy metal ions, replacing oil-based lubricants. The newly developed lubricant can perform effectively at elevated-temperature conditions as effective as ZDDP’s but are more water-soluble, and environmentally-friendly.

As the rubbing surface often operates in boundary lubrication, a boundary tribofilm often occupies the first few layers of the rubbing surfaces, leading to a three-body sliding system^6^. The friction and wear behavior of the contacting surfaces thus depends strongly on the tribofilm, which is essentially related to the chemical composition and fine structure of the film. Intense research has been done to investigate the tribological reactions of lubricant additives like ZDDP over the rubbing surfaces^4^,^5^. In the case of the chemical nature, this polyphosphate tribofilm has a thickness in the range of 50~150 nm and, consists of short chain phosphate in the bulk and a thin top layer of polymeric-like phosphate, with a composition in gradient distribution^4^. Different mechanisms of film formation involve, including thermal, hydrolytic, or oxidative decomposition of ZDDP^4^,^7^. However, those work mostly performed at the relatively lower temperatures. As the glass transition temperature (*T*_*g*_) of polyphosphate glasses is not elevated, about 200 °C^8^. Warren *et al*. assumed appearance of a viscous flow of the molten glass tribofilm at the asperity-to-asperity contacts contributing to the anti-wear action of ZDDP^9^, but no work is reported to verify this propose. Tieu *et al*. have investigated alkaline polyphosphate at rubbing steel surfaces with the operation temperatures over 600 °C, the result has shown a desirable performance on reducing wear and friction is achieved in hot strip rolling and elevated-temperature tribotesters in the laboratory, which is attributed to with the hierarchical structure at the contacting surfaces^10^,^11^,^12^. In this case, the polyphosphate tribofilm is around 100 nm thick. The boundary film formed at bearing surfaces of course involves material transfer from the counterbody, thus producing a self-organized layer on the top of the original surface. However, neither the chemical heterogeneity of the tribofilm formed at elevated temperatures nor its spatial distribution at the sub-micron level is understood with a high degree of certainty, which is technologically critical to exploit new material and lubricant, and for application-oriented studies in product evaluation and failure analysis.

Essentially, the boundary tribofilm includes a variety of components, in which many oil-soluble alkali metal salts were added to oil-based lubricants^13^,^14^,^15^, and alkaline elements have been found to survive within the tribofilm^16^,^17^. It has been found in our previous work that those alkaline metallic elements could prevent oxidation, and can also reduce friction and wear^12^. Furthermore, they have a high polar attraction toward metal surfaces, thus facilitate the formation of a thicker film with additives. However, there have been controversial views on the acting mechanism of alkaline metallic elements in reducing wear and friction. Some argued that, the addition of alkali metal salts produced the synergistic effect in improving the oxidation stability of lubricants but they did not have any anti-wear property^16^. In another view, the addition of sodium and potassium compounds in the Fe-Cr-C coatings, their toughness and abrasion resistance are greatly improved^18^. However, how the K/Na addition can resist abrasion is unknown. Furthermore, these alkali elements play important role in extreme pressure behaviors and further affect the performance of the tribofilm^13^,^14^,^15^. But very limited scientific literature with experimental evidence is available to explain the role of alkali metal compounds that especially present at elevated-temperature rubbing surfaces.

In this study, the fine structure and chemistry of the polyphosphate tribofilm itself is identified by means of multi-scale techniques. X-ray absorption near edge structure (XANES) profiling, Time-of-Flight Secondary Ion Mass Spectrometry (TOF-SIMS) three dimensional depth profiling and TEM (Transmission Electron Microscopy) observations of FIB (Focused Ion Beam) cross-section are applied to study the evolution of the oxygen states (phosphate and/or oxide) through the tribofilm, the distribution of phosphorus and alkaline metal elements within the film and the evolution of the polyphosphate chain length in the film with elevating temperatures. Subsequently, the correlation of performance with the interfacial reactions of polyphosphate is particularly discussed, as well as the role of the alkaline elements in tribochemistry.

## Results

### Friction and wear results

The alkaline polyphosphate lubricants reduce friction and wear significantly for all the lubricated steel/steel contacts, and the tribology performance is found to be temperature-dependent, the friction and wear results were shown in Table 1 12. As compared to that in dry condition, a significant reduction in the friction and wear was achieved for steel/steel pair by the presence of polyphosphate^10^,^12^, as shown in Table 1. In this study, great efforts will be made to characterize the fine structure and chemistry of the polyphosphate tribo-boundary film itself, to optimize the lubricity of the alkaline polyphosphate, and to understand the role of alkaline elements within the tribofilm.

### Focused ion beam (FIB) milling and TEM analysis

Initial surface elemental analysis via EDS tells us that sodium, phosphorus and oxygen elements mainly exist on the worn surfaces of steel specimens lubricated by polyphosphate while potassium is mainly concentrated at the interface region between the tribofilm and iron oxide layer, evidenced by TEM and EDS^10^,^11^,^12^. XPS analyses on the surface of lubricated steel have identified the shift of binding energy for the principal elements in lubricant as well as the relevant iron, iron or iron oxides are digested into the tribofilm under thermal and mechanical stresses, and the longer chain-length polyphosphate became short chain-length phosphate at the near surface^12^. Both the EDS and TEM observation indicated a boundary film on the worn surface as a result of tribochemical reactions between the contacting iron oxide scales and melting polyphosphate, and the film is around 120 nm thick^12^. It considered four regions of the tribo-interface from surface to the steel base in the cross-section view, little information about the tribofilm was obtained^10^,^12^.

**Figure 1:**
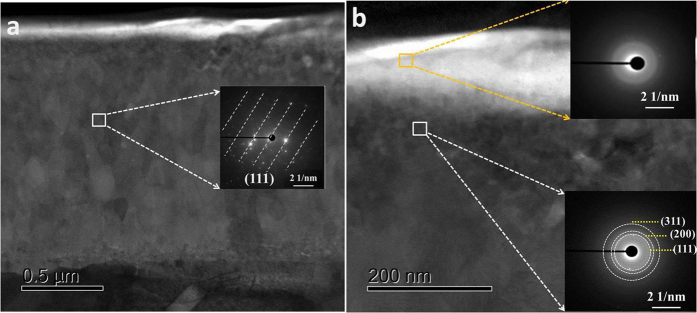
TEM images and the corresponding selected area electron diffraction patterns (SAED) of the tribofilm at 800 °C.

**Figure 2:**
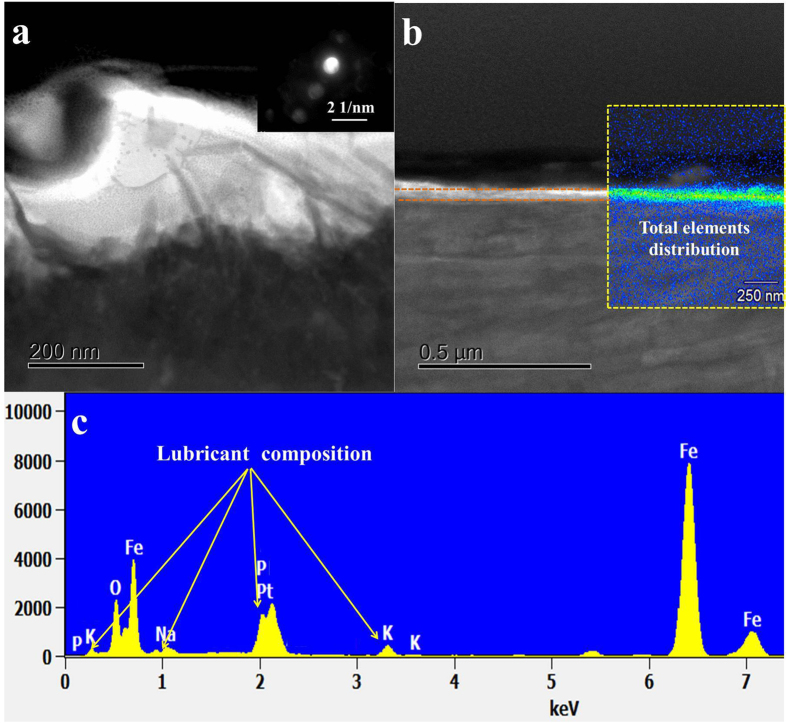
(**a**)TEM image and SAED pattern of the tribointerface at 800 °C, (**b**) FE-SEM image and total elemental distribution, (**c**) as well as the corresponding EDX.

Figures 1 and 2 showed the TEM images with different magnifications, electron diffraction (ED) patterns, and EDS spectra for the cross-section sample extracted by FIB from the worn steel surface. There is a boundary film adhering onto the iron oxide sublayer, the thickness of this film is comparable to the value obtained from ZDDP-derived tribofilms^3^, around 120 nm. As shown in Fig. 1a, the iron oxide interlayer is well-defined and had good adhesion to the steel base. The selected area electron diffraction (SAED) pattern shows the typical (111) plane of iron oxide (Fe_2_O_3_), suggesting the dominate iron oxides within the interlayer. The boundary film seems more featureless, non-uniform and porous in Fig. 1b, indicating an amorphous nature of polyphosphate. However, the SAED pattern from the position between the tribofilm and iron oxide reveals three concentric diffuse rings and a halo in the center. Accordingly, the *d*-spacing of the rings is 0.207 and 0.12 nm, which indicates short-range crystalline phosphate corresponding to (111), (220) and (311) respectively. When we get the SAED pattern that is very close to the surface, the SAED pattern demonstrates no feature and some diffraction spots can be distinguished vaguely, which suggests the top layer of tribofilm is dominantly amorphous. The boundary film is thus a nanocomposite with possible nanocrystals (Na (K)FeP_2_O_7_, FePO_4_, etc.) incorporated into the amorphous polyphosphate, suggesting the interaction between iron oxide and phosphate.

Figure 2a shows clearly an irregular edge resolution among the interface, which indicates the diffusion takes place between iron oxide and alkaline polyphosphate tribofilm. As for the inclusion of iron oxide in the tribofilm, it is probably due a quick removal of material during the running-in period before a protective film is built up, and some metallic wear debris are trapped at the sliding interface and then chemically and mechanically mixed in the tribofilm formation. The running-in process leaves a relatively rough surface which deforms under load while interacts with the wear debris and tribo-chemical products. This finally results in a non-uniform film thickness on the heated steel surface. Thus, a non-uniform polyphosphate tribofilm in white color can be observed on the SEM image in the cross-section view, as shown in Fig. 2b. It shows all the element distributions of alkaline polyphosphate lubricant from the overlaying process of all elements of lubricant, as shown in the illustration of Fig. 2b. The EDS image in Fig. 2c confirms a distinct film composition compared to that of the substrate, with a substantially higher sodium, potassium, iron, phosphorus and oxygen.

### SIMS/TOF-SIMS analysis

**Figure 3:**
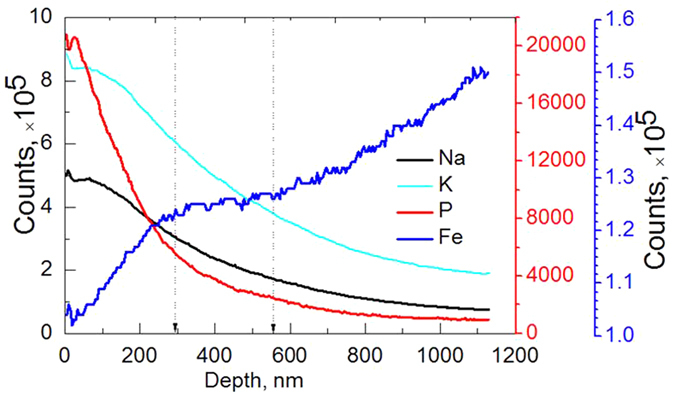
Depth profile of the composition in the worn steel disc at 800 °C by SIMS.

The chemical composition through the probing depth is determined by SIMS with the aid of Cs+ sputtering, as shown in Fig. 3, where the depth profiling is obtained from the worn steel surface at 800 °C. As the worn surface is very rough, the probing depth is roughly estimated, about 912.9 nm. A gradual change presents, a substantial amount of Na, P and K elements locates at the uppermost layer, and simultaneously the iron element increases steadily toward the substrate. Furthermore, the metallic iron is almost zero at the top surface, this views sodium (potassium) polyphosphate as the primary component at near surface with very little iron polyphosphate/iron oxide. The principal components of lubricant present as deep as 300 nm, i.e. sodium, potassium, phosphorus and oxygen, however, there is no clear edge resolution presenting between the boundary film and an intermediate layer. We believe that, strong diffusion occurs at the interface, the principal iron phosphate intermediate with small amount of sodium (potassium) within the interface formed, producing a heterogeneous nanostructure. Also, the SIMS well confirms TEM and SAED pattern in Fig. 2. The polyphosphate boundary tribofilm can accommodate oxide scale by the tribochemical reactions^3^,^19^. Especially alkaline phosphates are chemically reactive, once it presents at the heated steel surface, an inorganic crystalline or amorphous iron phosphate film generates quickly as an integral part of the metal surface. The existence of a certain amount of phosphorus at the transition further demonstrates the proposed structure. This resultant protective film is adherent, which is much less reactive to subsequent corrosion and oxidation than the original metal surface.

**Figure 4:**
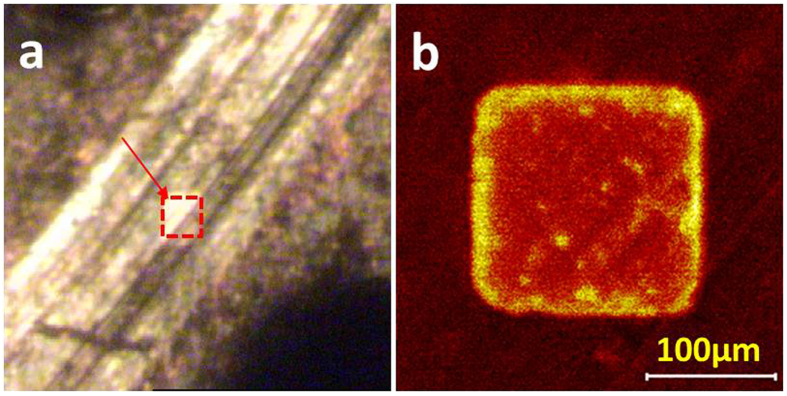
Surface morphology and analysis SIMS depth profile inside the wear track.

**Figure 5:**
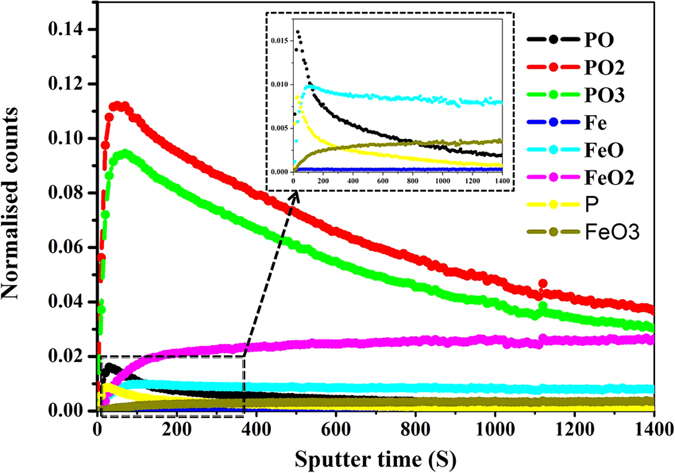
Depth profile of the composition in the worn steel disc at 800 °C by the dynamic TOF-SIMS.

TOF-SIMS imaging of elements and low-mass ions possesses the monolayer surface sensitivity, sub-micron resolution can be achieved. Figure 4 shows an image of the crater produced in the worn surface after the dynamic TOF-SIMS analysis. This displays the tribofilm covers some scratches visible on the worn steel surface after etching, indicating the tribofilm has been formed superimposed on the abrasive wear scratches during the wear-in period. The position of the analyzed area (100 × 100 μm^2^) is visualized in the crater. The crater has a rough depth of 150 nm. Different ions depth profiles of the molecular fragments (PO^−^, PO_2_^−^, PO_3_^−^, FeO, FeO_2_ and FeO_3_ and P^−^) extracted from an area of 100 × 100 μm^2^ inside the worn surface are presented in Fig. 5. It is observed that the molecular ions PO_2_^−^ and PO_3_^−^, typical characteristics of polyphosphate, show depth profile as similar as those observed for the distribution of P^−^ and PO^−^, with the maximum intensity at 90 ± 5 seconds of etching. Among them the TOF-SIMS depth profiles for PO_2_^−^ and PO_3_^−^ fragments are most intense at the worn surface, and then decrease rapidly with etching time in Fig. 5. As for the relevant iron species, they are showing the increasing intensity with depth in the surface and sub-surface region of the tribofilm followed by a stable FeO and FeO_3_ intensity among the interface, and the maximum intensity is at 100 ± 5 seconds for them. It is noted that, the depth profile in the maximum intensity is different between the P^−^ and Fe relevant species. This supports a diffusion layer existing between the boundary film and the oxide intermediate. Rossi *et al*. has imaged the adsorption and possible reaction of phosphate-type lubricant additives (zinc and sulfur free) on ferrous materials^20^.

**Figure 6:**
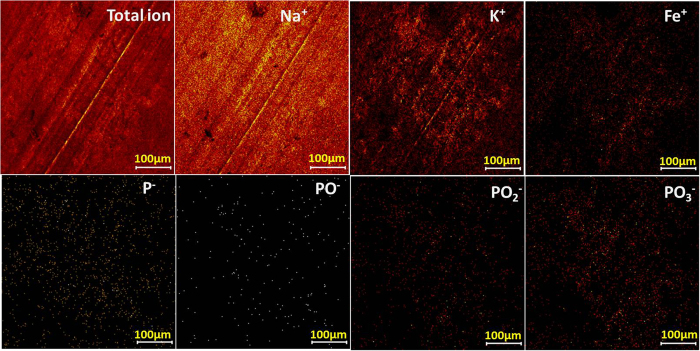
Positive and negative secondary ion and total ion signals obtained from the originally worn surface at 800 °C: Intensities are color-coded.

**Figure 7:**
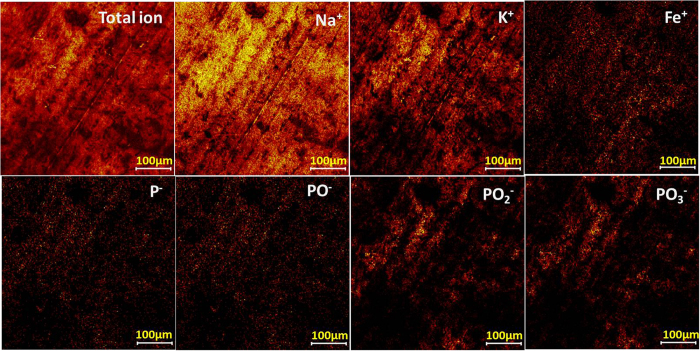
Positive and negative secondary ion and total ion signals obtained from the sputtered area within the worn surface at 800 °C: Intensities are color-coded.

TOF-SIMS further observes the main distribution of the molecular fragments on the origin worn surface as shown in Fig. 6. The fragments in the chemical are described by contrast, for example, a bright area indicates high concentration of a focused element. It shall be noted that, Na^+^, K^+^ and Fe^+^ are detected as the main ion in the positive ion spectra, and fragment ions of polyphosphate are detected in the negative ion spectra, i.e. PO_2_^−^ and PO_3_^−^. This again indicates an alkaline iron polyphosphate film is over the frictional surface, where Fe^+^ is normally thought to be from the counterpart steel ball. As the worn surface is very rough, this would cause a significant reduction in mass resolution, which resulted from an uneven electrostatic field on a rough surface. After etching, the concentration of those fragments increase and become more uniform in Fig. 7. According to the TOF-SIMS imaging of those ions, polyphosphate film is mainly covering onto the valley area in the worn surface and, this tribofilm is considered to be chemically inhomogeneous at the submicron level, which is attributed to the difference in contact.

**Figure 8:**
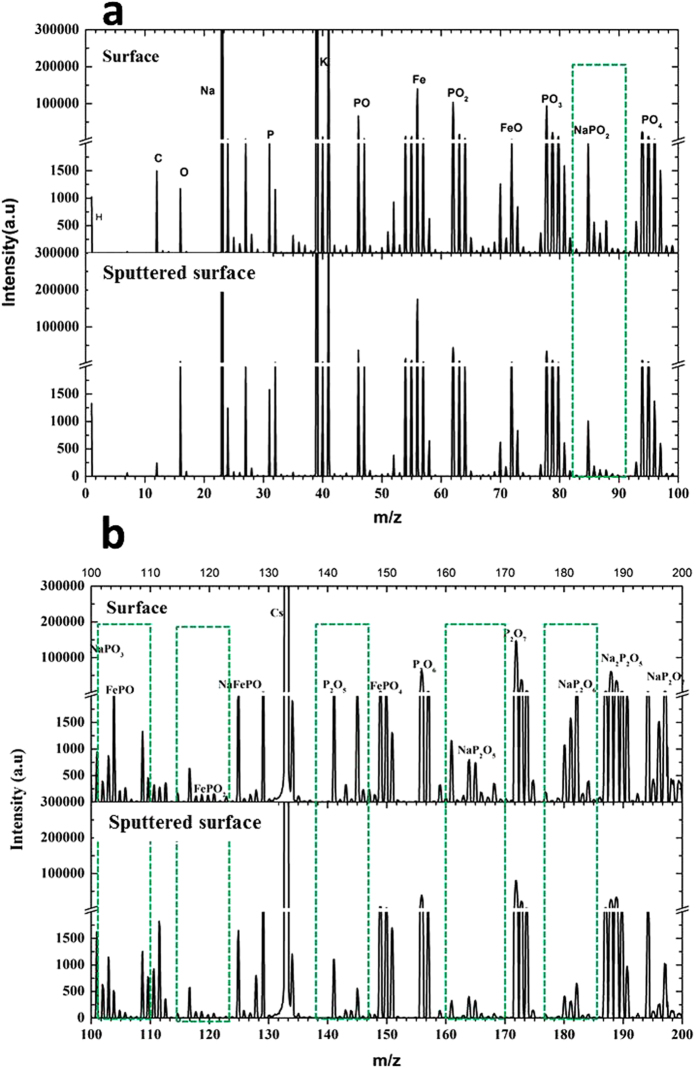
The TOF-SIMS spectra of the friction surfaces and the sputtered surface from: (**a**) 0~100 amu; (**b**) 100~200 amu.

TOF-SIMS analysis has the sensitivity to the first top monolayer, which permits a focus on changes of surface chemistry. The SIMS spectra for positive and negative ion in the mass range 0~200 amu of the friction surfaces at 600 °C, 700 °C, and 800 °C are obtained, respectively. The results are almost similar. All spectra show the presence of sodium, potassium, phosphate, and iron, in agreement with the XPS results^12^. To illustrate the major changes in chemical composition induced by friction, TOF-SMS spectra from the origin worn surface obtained at 800 °C and the correspondingly sputtered area are shown in Fig. 8, where the positive and negative ions are presenting. The major ion peaks in the mass spectra are assigned to the phosphate fragments PO^−^, PO_2_^−^, PO_3_^−^, and PO_4_^−^, which probably originate from the liner structure of polyphosphate. At higher masses a typical pattern for the sodium or iron phosphates could be identified, i.e., fragments containing up to two phosphorus atoms with a periodicity of PO_2_: NaPO_3_^−^, NaP_2_O_6_^−^, NaP_2_O_7_^−^, and FePO_2_^−^. This suggests a large amount of longer chain-length phosphate within the tribofilm, especially at high masses (i.e. NaP_2_O_7_^−^)^21^. Additional peaks can be found to be attributed to iron oxide and iron hydroxide (FeO^+^, FeOH^+^), and their isotopes. These data proves that a tribo-chemical reaction between alkaline polyphosphate and iron oxide occurring to produce iron orthophosphate, iron and sodium mixed phosphate and sodium pyrophosphate. The major difference in the mass spectra between the as-obtained worn surface and the sputtered area within the worn surface is that, the characteristic ion at m/z = 85, 141,165 and 180 the periodicity of PO_2_ of alkaline polyphosphate has a lower relative intensity and that the ions almost have disappeared. And the peak at m/z = 110 well corresponding to the FePO^−^ fragment shows an increasing intensity, which means the appearance of iron phosphate compounds at the intermediate of the tribofilm. The tribochemical interactions due to the sliding contact causes changes in the surface chemistry. Polyphosphate is chemically adsorbed onto the iron oxide, forming iron phosphate under the conditions of loaded contact, for example.

**Figure 9:**
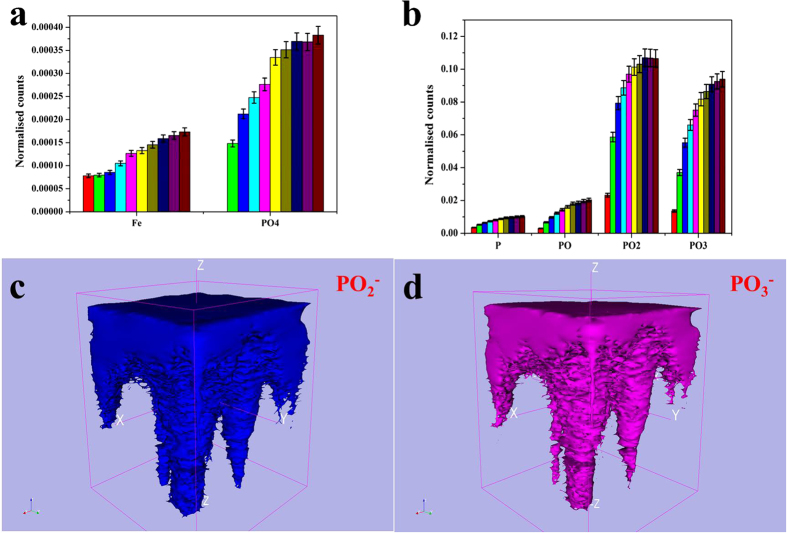
(**a**,**b**) Counts of fragments distribution with the etching duration of 10 s, (**c**,**d**) TOF-SIMS 3D depth profiles of the PO_2_^−^ and PO_3_^−^ fractions inside the sputter crater of the worn surface at 800 °C.

Furthermore, 2- and 3-D TOF-SIMS depth profiles are performed to investigate the boundary layer formation on the steel disc surfaces at temperature of 800 °C. Figure 9a,b depict the views of the PO_2_^−^ and PO_3_^−^ fragments obtained from the etching crate within the worn surface, as well as Fe and PO_4_^3−^. Those fragments increased in gradient trend, however, after 8s etching the PO_2_^−^ group becomes decreasing. This indicates long chain polyphosphate is reduced and, iron phosphate compound increases across the tribofilm. The comparison of the intensities of those fragments in Fig. 9a,b indicate that, metaphosphate fragment is dominant at the top layer, with a substantial amount of orthophosphate appearing. The images in Fig. 9c,d show the lateral distribution of the PO_2_^−^ and PO_3_^−^ signals. During the etching process PO_2_^−^and PO_3_^−^ remain at a high level on and position near the top surface region of tribofilm at the outermost surface of the worn steel disc.

### XANES analysis

Correlation of structural change with the performance of ZDDP-derived tribofilm has been analyzed by XANES^22^. Chain-length of polyphosphate varies with temperatures and shear stresses, for example^23^. XANES technique has two modes: total electron yield (TEY) and fluorescence yield (FY) modes. The TEY mode has a surface sensitivity of ~5 nm, while the FY mode probes >50 nm^22^, thus the simultaneous TEY and FY analysis can provide complementary information about the chemistry at the uppermost surface and in the bulk of the tribofilm.

**Figure 10:**
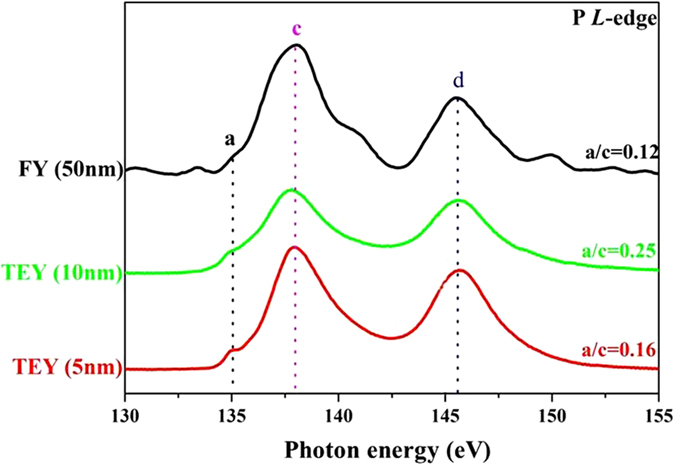
P *L*-edge XANES spectra of the polyphosphate tribofilm in fluorescence yield (FY) and total electron yield (TEY) modes, respectively. TEY (10 nm) spectrum is obtained from the area after etching. The peak ‘a’ is typical characteristic of polyphosphate glass. The height of this peak (compared with peak c) is related to the polyphosphate chain length.

Figure 10 shows the P *L*-edge XANES spectra on the wear track in TEY and FY modes, where the *L*-edge has a narrower linewidth and better photon resolution, thus the P *L*-edge is more sensitive to determine the chemical and structural characteristics than that of the *K*-edge. It is reported that, the probing depth is ~5 nm for the P *L*-edge and ~25 nm for the P *K*-edge, respectively^22^. The general shape of the spectrum is characteristic of the phosphate bonding and the peak ‘a’ is characteristic of the polyphosphate structure^24^. The relative intensity of the peak ‘a’ and peak ‘c’ is known to be as the function of the polyphosphate chain-length. For example, the value of a/c for orthophosphate, pyrophosphate, and polyphosphate is 0.13, 0.26 and 0.75, respectively^24^. Of course, a range of chain lengths occurs in these tests, as the peak ‘a’ is not sharp compared with the standard. As the relative height of peak ‘a’ decreases significantly from the top surface (TEY) into the bulk of the film (FY), this allows us to know the average chain length in the bulk is smaller than on the surface. After etching, the a/c ratio is 0.25, which indicates a relatively longer chain length polyphosphate is primarily located at the depth of 5 nm from surface. Moreover, not all FY data are reported since the signal is very weak. As the probing depth of the FY technique is ~50 nm, the poor signal implies that the antiwear film formed within the wear track is not uniform and thin, and the thickness of the region profiled is believed to be no more than 50 nm.

**Figure 11:**
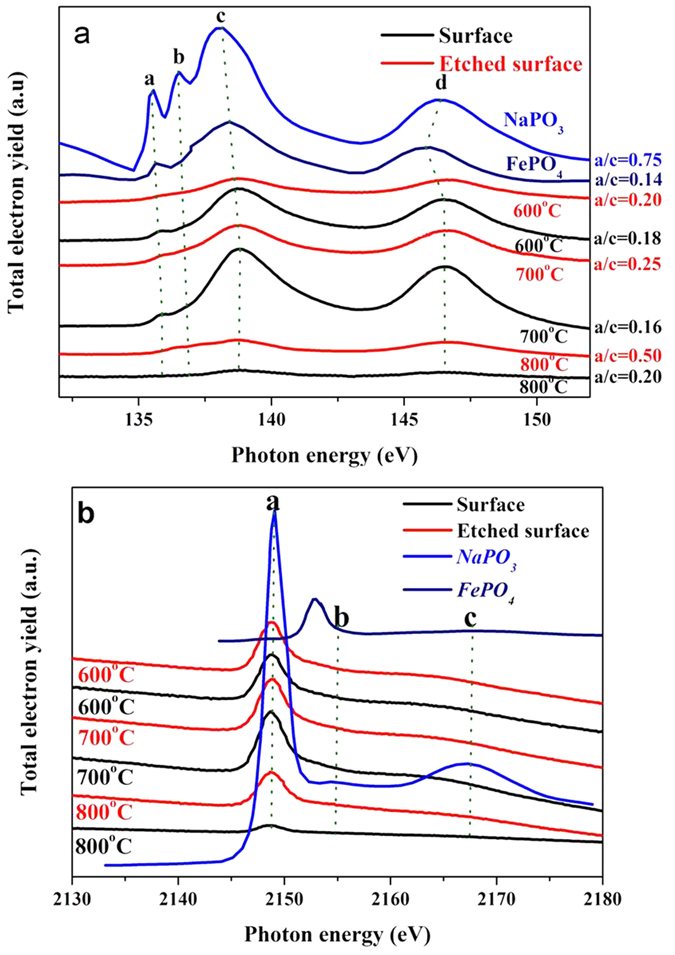
P *L*-edge (**a**) and *K*-edge (**b**) XANES spectra of tribofilms at 600, 700, and 800 °C.

Phosphorus *L*- and *K*-edge XANES spectra of tribofilm at 600 °C, 700 °C and 800 °C recorded in TEY mode, are shown in Fig. 11a,b, where the relevant model compounds are presented for comparison. These spectra are obtained from the originally worn surface and the etched surface within the wear track. Four typical peaks present in the P *L*-edge spectra, which provide the evidence of the electron transition from the 2p orbitals to unoccupied higher level *s* and *d* orbitals^24^, as shown in Table 2. It can be noted that, the peak positions of in all spectra are highly similar to the positions for the model NaPO_3_, as shown in Table 3. In particular, the position of ‘a’ peak in the tribofilm is much closer to peak of NaPO_3_ (~135.6 eV) than peak for FePO_4_ (~135.3eV). Further, lack of the pre-edge peak implies that there is a low content of iron phosphate at the near surface, thus the major cation in the near surface region of these films are Na^+^ rather than Fe^3+^, which confirmed the TOF-SIMS analysis in Figs 5, 6, 7, 8, 9. Interestingly, the a/c values are nearly the same for the lubricated surface at 600 and 700 °C, while the worn surface at 800 °C had a higher a/c value of 0.2. This is also confirmed by the P *L*-edge XANES spectra from the etched surfaces. The etched surface in the wear track has a value of 0.25 at 700 °C and 0.20 at 600 °C respectively, while the surface at 800 °C displays a value of 0.50. At elevated temperature, the chain-length of polyphosphate increases, but is lower than the reference NaPO_3_ compound, during friction sodium polyphosphate of lubricant decomposes partly. As compared to the top layer of surface, the etched surface is dominated with relatively longer chain-length polyphosphate, especially at the temperature of 800 °C. Therefore, the uppermost surface mainly comprised of longer chain-length polyphosphate after etching, however, a short chain-length orthophosphate existed in the whole tribofilm. There is no contradiction with the analysis in Fig. 11, which is possibly due to the post-oxidation after testing. This is associated with the nature of polyphosphate and the cross-linking or shortening of the polyphosphate network induced by the shearing stress^25^.

The spectra in Fig. 11b is characteristic of typical P *K*-edge XANES spectra for phosphates. The explanation of the absorption features has been expressed elsewhere^24^. All spectra are the similar patterned, the tribofilm chemically thus resembles with the model compound (NaPO_3_), which can be observed from the peak positions in Table 3. Normally, they comprise one main peak ‘a’ and several weak peaks like ‘b’ and ‘c’, their attribution can be found in Table 2. On the other hand the peak ‘b’ is very weak in glass or amorphous state than in crystalline phosphate in the P *K*-edge spectra, thus the feature of polyphosphate is amorphous within the tribofilm in this study. In the phosphate tribofilm, although an amount of iron species is digested into the polyphosphate matrix, the P atom remains the same coordination with four oxygen atoms. This will probably cause the shift in absorption energy. However, the energy shifting is invisible as shown in Fig. 11b.

**Figure 12:**
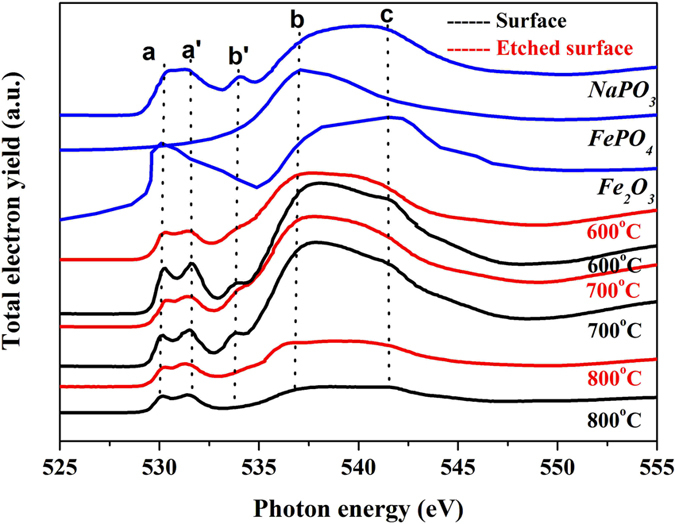
O *K*-edge XANES spectra of tribofilms at 600, 700, and 800 °C.

The O *K*-edge XANES spectra in Fig. 12 allows us to identify the cations of phosphate, especially can determine the amount of iron presenting within the tribofilm^25^. In general, the iron oxides shows the splitting peaks ‘a’ and ‘a’, as well as FePO_4_ and NaPO_3_. The corresponding peak positions are shown in Table 3. After the rubbing tests some iron oxide scales will be digested into polyphosphate matrix and, substitute part of Na_2_O, this indicates phosphate compounds containing sodium and iron elements existing within the tribofilm from the evidence of the splitting peak in Fig.12. Noted that intensity of peak ‘b’ decreases with the elevating temperature, which relates to the variation in the coordination of iron ions and phosphate ligands during the process of depolymerization and simultaneous cross-linking. Though incorporation of iron contributes to the energy shift from O1 s to the Fe3d-O2p hybridized band, the energy shift is not discernable, since the content of Fe-O-P bonding is lower than that of the bridging oxygen (P-O-P) within the tribofilm. Especially, the intensity of peak ‘b’ in the etched surface is higher than that of the uppermost surface, this supports the view that the iron content increases from the surface to the depth.

**Figure 13:**
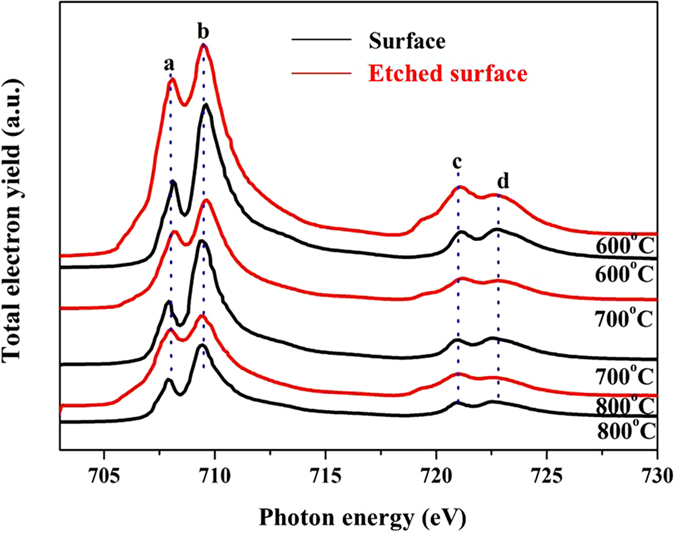
The Fe *L*-edge XANES spectra of tribofilms at 600, 700, and 800 °C.

The Fe *L*-edge XANES spectra in Fig. 13 clearly shows two doublet types, *L3* and *L2* edges, corresponding to electron transition from 2p3/2 and 2p1/2 energy levels respectively^26^,^27^, as shown in Table 2. The energy separation between the peaks in the *L*3 edge is about 1.6 eV in the tribofilm. If Fe in octahedral coordination in the tribofilm, t_2g_ orbitals could accommodate 6 electrons while e_g_ orbitals could contain a maximum of 4 electrons, as Fe^3+^ has 5 electrons in its 3d level, both t_2g_ and eg orbitals can receive electrons from the 2p level, therefore a doublet appeared in the absorption spectra. However, Fe^2+^ had 6 electrons in its 3d level and only eg orbital could accommodate electrons from 2p level, so Fe^2+^ showed no doublet structure. If Fe in tetrahedral coordination in the tribofilm, only t_2g_ orbital is able to accept electrons from 2p level regardless of Fe^2+^ or Fe^3+^. In this study, the main Fe^3+^ in octahedral coordination is dominant within the tribofilm, which is consistent with published works^23^,^28^. As Fe^3+^ ions in polyphosphate are preferably bonding in octahedral coordination^29^, FePO_4_ normally shows a very narrow and sharp peak ‘a’, while Fe_2_O_3_ has the characteristic splitting of the *L*2 edge. As compared to the Fe *L*-edge XANES spectra from FePO_4_ and Fe_2_O_3_, it can be figured out that both iron oxide and iron phosphate are presenting in the tribofilm. Iron ions do participate in process of tribofilm formation, are most important as network modifier-formers in the polyphosphate tribofilm. During friction the combined effect of high pressure and temperature will facilitate the cross-linking bonding of iron atoms and non-bridging oxygen atoms, increasing the elastic modulus and nanohardness of the tribofilm^30^.

**Figure 14:**
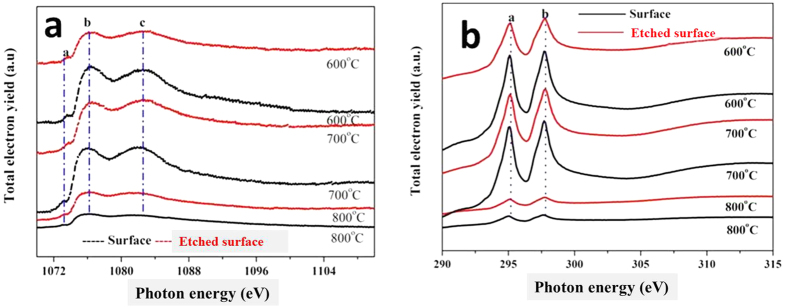
(**a**) Na *K*-edge and (**b**) K *L*-edge XANES spectra of the lubricated surfaces at 600, 700 and 800 °C.

Furthermore, Fig. 14a displays the Na *K*-edge and K *L*-edge XANES spectra of the lubricated surfaces at 600, 700 and 800 °C, which can determine the coordination state of the specific element in materials^24^,^30^. For the Na *K*-edge spectrum, if the doublet (b and c) is wide and has low frequency oscillations at higher energies, Na atoms are not located in the periodic medium/long distance of phosphate. In this study, Na atoms are in amorphous or glassy state environment, its disorder degree increased with temperature. It is further noted that the disorder degree within the etched surface is more than that on the uppermost surface. Normally Na and K are the archetype modifiers in glass or amorphous material. As for the role of Na in tribofilm, iron oxide is chemically digested into polyphosphate tribofilm, the sodium atoms not only serve as the modifier to compensate the charge during the tribofilm evolution, and also play the role of flux modifier to regulate the viscosity of melt during friction. Meanwhile K is similar to sodium, but its ionic mobility is not better than that of Na because of the larger ionic radius. The sharp resonance (doublet) in Fig. 14b locates at 296 and 298 eV can be assigned to the *L*2, 3-edge absorption of K, which is consistent with the inclusion of inorganic impurities associated with the highly oxidized components, e.g. K_2_O into a P_2_O_5_ glass of tribofilm^31^. As reported, amorphous phosphate having the layered structure is an excellent host that can allow alkaline ions (K^+^ and Na^+^) to be inserted or transported between the layers at high temperature^32^. It is believed that alkaline ions have the important effect on the tribofilm evolution, however, the effect of Na/K in reducing wear and friction cannot be distinguished from each other.

## Discussion

### Tribofilm acting mechanism

The friction and wear performance of tribofilm strongly correlates with their chemical composition and structure, which relates to the ability of polyphosphate glasses to digest iron oxides by the tribochemical reaction^4^. According to the hard and soft acids and bases (HSAB) principle, the polyphosphate is generally hard bases, orthophosphate in the lubricant is chemically hard as well; Fe^3+^ is a harder Lewis acid than Na^+^ and K^+^. Therefore, polyphosphates of the lubricant energetically reacted with iron oxides under the combined effect of temperature and shearing stress, especially at elevated temperatures the acid-base reaction and the cation exchange are very favorable^4^. For example, the chemical reaction of sodium polyphosphate with Fe_2_O_3_ shall be as follows
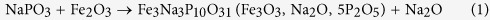


Reaction (1) is an illustration of the possible reacting process between iron oxide (as Fe_2_O_3_ in this case) and alkaline oxide (e.g. Na_2_O). The digestion of the iron oxide into the polyphosphate glass has been confirmed by TOF-SIMS and XANES analysis in this study. Apart from the fragment ions originating from the lubricants, complex compounds such as Fe_2_O_3_, FePO_4_, and NaFeP_2_O_7_ are clearly detected in the mass spectra in Figs 7, 8, 9, which are proposed to be product by the tribochemical reactions^33^. Fe *L*-edge XANES spectra also confirms the tetrahedral and octahedral coordination of Fe^3+^ within the tribofilm in Fig. 13. The friction process often initiates such reactions by high shear stress and high temperature up to 1300 K^4^,^19^. In this case, this reaction occurs as expected, since the polyphosphate chain becomes shorter and iron phosphate generates, as evident from the evidence of P *L*-edge and Fe *L*-edge XANES spectra in Figs 11 and 13. Moreover, more generally some iron oxides exist within the tribofilm in Figs 12 and 13, which is consistent with the TEM&SAED analysis in Fig. 1.

### The role of alkaline elements

As for the chain length of polyphosphate tribofilm derived from ZDDP, it decreases in a gradient change from the surface to the depth, while the tribofilm consists of short chain-length polyphosphate in the bulk^4^,^19^. According to TOF-SIMS images in Fig. 9, there is probably a gradient layered structure within the elevated-temperature tribofilm as ZDDP’s^33^: short-chain Fe/Na polyphosphates in the bulk and, a longer chain-length polyphosphate at the top. However, XANES analysis in Figs 10 and 11 indicate the appearance of shorter chain-length polyphosphate at the uppermost surface. After a ~5 nm thick film was etched, the etched surface is made of relatively long-chain polyphosphate, while the bulk of tribofilm is short chain-length polyphosphate. In this study, the friction testing was done at elevated temperatures where the severe oxidation process of course involves chemical reactions of polyphosphate with oxygen. During friction alkaline polyphosphate have to react with oxygen exposure, more oxygen will be adsorbed through the chemical reaction and further oxygen penetration will be inhibited, producing more non-bridging P-O bonds and thus the chain-length of polyphosphate becomes much shorter. Both temperature and shearing stress are the dominant factors influencing the chain-length of polyphosphate, where the temperature is mainly considered in this work. The comparison of friction and wear results in Table 1 indicates the performance in reducing wear and friction is better at 800 °C, the chain-length of polyphosphate is longer accordingly as shown in Fig. 11a, which is consistent with the results of ZDDP’s^4^,^19^,^34^. As XANES analysis shown in Fig. 11a, the a/c ratio increases with increasing temperature, especially for the etched surface. Under temperature and shearing stress, the addition of iron ions within polyphosphate involves the exchange of Na^+^ with Fe^3+^, which permits more negative charges to balance the reaction, and as a consequence the shortening of the chain length occurs. Therefore, short chain-length mixed Fe/Na polyphosphates lies in the bulk of the tribofilm.

For the tribofilm at elevated-temperature interface, the stable structural arrangement is very important for the chemical and mechanical characteristics of polyphosphate during friction. Polyphosphate is normally regarded as the amorphous network consisting of long chains of PO_4_ tetrahedral interconnected by bridging oxygen atoms^35^. Polyphosphate matrix can accommodate modifier cations easily, which will cause structural evolution among the phosphates glass. The interaction between the metal cations and the nonbridging oxygens of polyphosphate contributes to the improvement in physical and chemical properties. For example, the doped sodium phosphate glass becomes very hard and elastic. As the iron (III) ions are in tetrahedral and octahedral coordination or distorted octahedral coordination, during friction the reaction of iron oxides (Fe^3+^) with polyphosphate matrix will produce more strong Fe-O-P bonds and more P_2_O_7_^4−^ groups, and simultaneously the degree of network increases, as compared to alkaline polyphosphate. This will make the polyphosphate network much tighter and more cross-linked, which is believed to be responsible for an improvement of chemical and mechanical stability of polyphosphate tribofilm, resulting in reducing wear.

Alkaline compounds have been applied as the additive in engine oils at ambient temperatures, which are helpful to the tribofilm formation at ambient temperatures due to their chemical affinity with the metal surface^13^,^14^,^15^,^16^,^17^. In this study, alkaline polyphosphate is the high-temperature lubricant, in which alkaline elements (Na^+^ and K^+^) are included; they normally coordinates 1.5–2 O atoms in the polyphosphate and are the archetype modifiers. They can break the bridging bonds P-O-P, generating negatively charged oxygens^36^. During the synthesis of sodium iron phosphate glass incorporation of iron ions into sodium polyphosphate preferably results in a layered arrangement^37^,^38^, where alkaline ions interact ionically with surrounding oxygens, setting themselves rather loosely in the holes of the amorphous network and keeping the balance of structural evolution. Thus, under the effect of friction and heating, the reaction of iron ions with sodium polyphosphate will produce sodium iron phosphate compounds with medium-range ordering within amorphous polyphosphate matrix, in which alkaline ions are located at the network of the phosphate compounds. As alkaline ions prefer to transport within amorphous phosphate compounds (e.g. FePO_4_)^34^, this will combat lattice distortions under shearing stress and elevated temperature conditions. As a result, in this study the tribofilm embodies the lubricating ability of layered lubricant similar to graphite and MoS_2_. Moreover, alkaline elements (Na^+^ and K^+^) are the powerful melting agents. Na and K species will lower the viscosity of melt at elevated temperatures, which reduces friction at the hot sliding surfaces.

As for Na^+^ and K^+^ ions of polyphosphate, the composition of Na^+^ in the solution is much higher than that of K^+^ in the formulation of lubricant. Due to the larger ionic radius of K, most of K^+^ is not likely to enter into the layered polyphosphate matrix and, would diffuse into the depth along the crystalline gap. This is why K was reported to be concentrated at the interface of tribofilm and oxide base^12^. The addition of alkaline elements increases the toughness of Fe-Cr based alloy^18^, thus it is believed that such diffusion of K^+^ will modify the mechanical property of tribo-interface in this study. Moreover, K is chemically active and has a high affinity to the metal surfaces, it thus yields the enhancing adhesion of tribofilm to iron oxide substrate. In order to understand the exact role of Na/K ions at the hot rubbing surfaces, further nanometer-scale modelling work by molecular dynamics calculations shall be done to reveal the structural dynamics of sodium (potassium) polyphosphate under pressure and temperatures.

**Figure 15:**
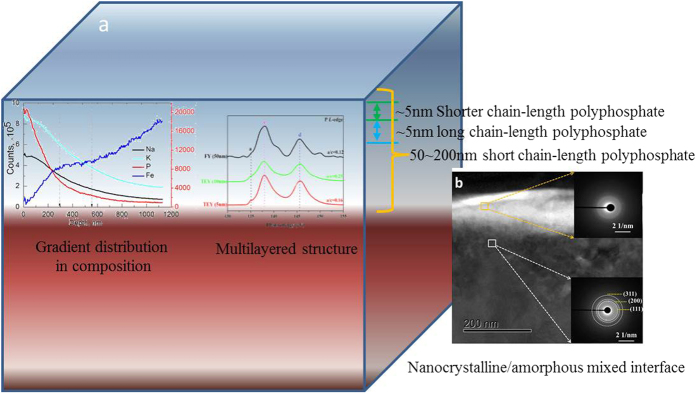
Schematic of the structure within the elevated-temperature tribofilm.

Accordingly, the schematic of the tribofilm is shown in Fig. 15. The tribofilm has a pronounced layered structure and gradient distribution in composition. The tribofilm derived from ZDDP has a two-layered structure whereas the tribofilm in this work consists essentially of ~5 nm shorter chain-length polyphosphate on the top layer, ~5 nm long chain-length polyphosphate at the sublayer that adheres onto the iron oxide substrate through a short chain phosphate. As the iron (III) ions are in tetrahedral and octahedral coordination, the reaction of iron oxides (Fe^3+^) with polyphosphate matrix will increase the degree of cross-linking bonding, causing an improvement in mechanical strength of tribofilm. Na^+^ and K^+^ alkaline elements are the charge compensators that keep the structural balance of tribofilm. They are the archetype modifiers, during friction the iron ions in combination with alkaline elements prefer to form the compounds with a layered structure. Moreover, they are also the viscosity modifiers for melt lubrication at elevated temperatures. Thus this tribofilm enables alkaline polyphosphate to function as the friction and wear reducer as well as the oxidation barrier.

## Conclusion

A multi-scale characterization approach is applied to determine the fine structure and composition distribution within the boundary film on the hot steel surfaces when alkaline polyphosphate is used as a lubricant. Analysis by TOF-SIMS and XANES indicate that the tribofilm has a varied thickness ranging from 50 to 120 nm. It shows different chain lengths of polyphosphate and has a gradient change in composition. However, the top layer of about 5 nm consists of a shorter chain-length polyphosphate; the sublayer (~5 nm) is composed of relatively long chain-length polyphosphate that adheres onto iron oxide steel base through a short chain phosphate. At higher temperatures, the chain-length of polyphosphate increases, the reduction of friction and wear is better for the steel/steel pair at 800 °C. The tetrahedral and octahedral coordination of iron ions makes the polyphosphate more cross-linking and improves the mechanical properties of the tribofilm. This contributes to wear reduction at hot rubbing surfaces. It is proposed that the layered structure and alkaline elements especially Na^+^ within the tribofilm is responsible for the lubricity of polyphosphate loaded contacts at the elevated-temperature, while K^+^ is helpful to improve the adhesion of tribofilm on iron oxide base and to modify the mechanical property of tribo-interface.

## Materials and Methods

A formulation of elevated-temperature lubricant consists essentially of sodium polyphosphate (NaPO_3_) and monopotassium phosphate (KH_2_PO_4_) in the mass ratio of 3.75:1, where the distilled water is the solvent. Two chemicals are grade of analytical reagent, which can be purchased from Sigma-Aldrich. In this study, sodium polyphosphate has the long chain-length structure.

Tribological tests are done using a ball-on-disc sliding configuration on a UMT2 Multi-Specimen Tribotesting System. Interstitial-free steel (IF) steel discs are used as the sliding/rubbing against chromium steel (Cr15) balls. The testing conditions are a rotation speed of 0.1 m·s^−1^, a normal load of 10 N (maximum Hertzian contact pressure 0.87 GPa). The testing temperature is 600, 700 and 800 °C, respectively. The lubricants are dropped onto the sliding surface by an external lubrication system with the flow rate of ~0.02 ml·s^−1^. The friction coefficient is measured and recorded automatically. The wear volume of the disc is measured and calculated using a Hommel T1000 profiler, the wear loss of the corresponding ball is calculated from the measured radius of the wear scar from SEM images.

**Figure 16:**
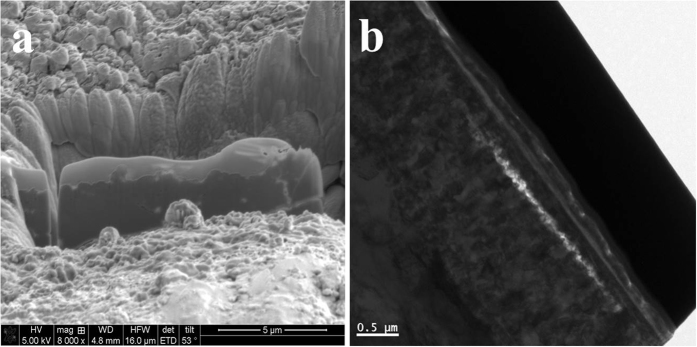
(**a**) FIB images of the tribofilm layer formed within the wear track at 800 °C and (**b**) the whole view of the tribointerface in cross-section view.

TEM (JEOL JEM-ARM200F aberration-corrected Scanning Transmission Electron Microscope, TEM) with Electron Diffraction, and SEM (JEOL JSM-7001F Field Emission Scanning Electron Microscope) with Electron Dispersive Spectroscopy (EDS) are used to measure the film thickness, visualize the nanostructure, and identify the crystalline phase within the tribofilm. The TEM samples are prepared using a FEI Nova 200 Dual-beam Focused Ion Beam (FIB) system with a Ga source with a fine beam current of 100 pA to extract a thin cross-section of the near-surface zone from the wear track of the specimen (15 μm ×15 μm). For example, Fig. 16 shows the area after a cross-section sample is extracted and a low-magnification TEM image of the overall view. A thin layer of Pt is deposited onto the wear track prior to the FIB lift-out procedure to protect the surface structure. A hierarchical structure has been observed through the cross-section of sample^14^.

This work also involves two coupling techniques: X-ray absorption near edge structure (XANES) and Time-of-Flight Secondary Ion Mass Spectrometry (TOF-SIMS), which will provide complementary information on the chemical species, bonding states and local geometrical environment within the tribofilm formed during the tribochemical reactions. XANES spectra at the P *L*-edge, P *K*-edge, Fe *L*-edge, K *L*-edge, Na *K*-edge, and O *K*-edge are recorded in order to fully characterize alkaline polyphosphate tribofilms in view of the photon energies. XANES analysis is carried out at the soft X-ray spectroscopy beamline of the Australian Synchrotron in Melbourne. The pressure of the vacuum chamber is maintained at better than 5 × 10^−9^Torr. This beamline is equipped with a plane grating monochromator that could provide 10^11^~10^12^ photons/s at 200 mA at the *K*-edge of the required element with a resolving power better than 10^4^. The beamline energy is 2800 ev with the photon resolution ≤ 0.2 ev. The analysed area is 1×1 mm^2^. All XANES spectra are taken simultaneously with total electron yield (TEY) and fluorescence yield (FY) modes. The maximum analysis depths at the L-edge are about 5 nm and 50 nm for TEY and FY, respectively. The photon energy scale for the Grasshopper monochromator is referenced to the lowest P *L*-pre-edge peak of sodium polyphosphate (NaPO_3_), at 136.5 eV. The sample surfaces are further cleaned by Ar^+^ sputtering for 60 s after the first XANES collection, and a film of approximately 10 nm is etched away.

Secondary ion mass spectrometry (SIMS) and Time-of-flight secondary ion mass spectrometry (TOF-SIMS) measurements are carried out at University of Western Sydney (UWS) and University of South Australia (UniSA), respectively. The SIMS system is equipped with a Cs primary ion beam with the total impact energy of 16 kV and a low-energy flood gun with an acceleration voltage of 20 eV for charge compensation. The Cs^+^ current is 6 nA. Mass spectral data is collected for the optimized electron spot size dimension for chemistry composition and structure during data collection and imaging. The chosen pixel size is 2048×2048 over a 180 ×180 μm^2^ region. The depth of the crater on specimen is 912.9 nm. Additionally, TOF-SIMS experiments were performed using a PHI TRIFT V nanoTOF instrument (Physical Electronics Inc., Chanhassen, MN, USA) at UniSA, which was equipped with a pulsed liquid metal 79 + Au primary ion gun (LMIG), operating at the energy of 30 keV. Dual charge neutralisation was provided by an electron flood gun (10 eV electrons) and 10 eV Ar+ ions. Experiments were performed under a vacuum of 5 × 10^−6 ^Pa or better. Positive and negative ion images were obtained using ‘unbunched’ Au1 instrument settings, typically using a 500 × 500 μm^2^ raster. Single gun Au1 negative SIMS depth profiles were recorded using a 50 × 50 μm^2^ analysis raster within a 150 × 150 μm^2^ sputter raster. Sample spectra, images and depth profiles were processed and interrogated using WincadenceN software (Physical Electronics Inc., Chanhassen, MN, USA). A pulsed low energetic electron gun is used for charge compensation. All spectra are calibrated using peaks in the spectra with well-known composition and masses. The sample surface are gently sputter cleaned for 20 s using the Ga ion beam and a sputter raster of 150 × 150 μm^2^ (1~2 nm is etched away), and the spectra in composition is obtained.

## Additional Information

**How to cite this article**: Wan, S. *et al*. Chemical nature of alkaline polyphosphate boundary film at heated rubbing surfaces. *Sci. Rep.* **6**, 26008; doi: 10.1038/srep26008 (2016).

## Figures and Tables

**Table 1:** Summary of friction and wear results^14^.

**Parameters**	**Condition**	**600** °C	**700** °C	**800** °C
Wear loss of disc (mm^3^)	unlubricated	0.103	0.269	0.476
	Lubricated	0.0787	0.1159	0.188
Wear loss of ball (mm^3^)	unlubricated	0.0476	0.0638	0.153
	Lubricated	0.0255	0.0309	0.0798
Coefficient of friction (μ)	unlubricated	0.482	0.458	0.435
	Lubricated	0.232	0.194	0.199

Note: The error is no more than plus or minus 5%.

**Table 2:** Peak assignments of the P *L*-and *K*-edge, O *K*-edge and Fe *L*-edge XANES (FY) spectra of tribofilms.

**Parameters**	**Peak**	**Assignment**^26^,^27^
P *L*-edge	a and b	the electron transition of the *2p*-electron to the a1* molecular orbital
	c	the electron transition of the *d* orbital to a *p*-like t2* orbital due to the blend of *p* and *d* atomic orbitals
	d	*d*-like shape resonances both in crystalline and amorphous phosphates
P *K*-edge	a	The electron transition from the 1s core level to the unoccupied t2* (*p*-like) anti-bonding orbital
	b and c	shape resonances or multiple scattering
O *K*-edge	a and a’	the transition from the O1s to 2p states that are hybridized with the partially filled Fe 3d band that are separated by the ligand-field splitting (O1s→Fe3d-O2p)
	b’	the O1s to O2p transition that is hybridized with either the 2s or 2p of phosphorus (O1s→P2sp-O2p)
	b and c	backscattering of the photoelectron from the next coordination shell^39^
Fe *L*-edge	a and b	the electron transition from O 2p to unoccupied Fe3d orbitals (*L*_*3*_- edge), where peak a is due to transitions from Fe2p3/2 to the Fe3d-O2p hybridized bands, and b is assigned to the Fe 3d (t_2g_)-O2p and Fe 3d (e_g_)-O2p hybridized bands^27^
	c and d	set of doublet in the *L2* - edge

**Table 3:** Peak positions of the P *L*-and *K*-edge, and O *K*-edge XANES (FY) spectra of tribofilms at different temperatures and model compounds.

		**P** ***L*****-edge** (**eV)**				**P** ***K*****-edge** (**eV)**			**O** ***K*****-edge** (**eV)**				
**Parameters**		**a**	**b**	**c**	**d**	**a**	**b**	**c**	**a**	**a’**	**b’**	**b**	**c**
600 °C	S	~135.6	~136.7	~138.7	~146.5	~2148.7	~2155.1	~2167.2	~530.3	~531.6	~533.9	~537.7	~541.5
ES	~135.7	~136.6	~138.6	~146.4	~2148.9	~2155.1	~2167.4	~530.2	~531.5	~533.8	~537.2	~540.8	
700 °C	S	~135.8	~136.8	~138.8	~146.5	~2148.7	~2155.1	~2167.2	~530.1	~531.5	~533.8	~537.8	~541.5
ES	~135.7	~136.7	~138.8	~146.5	~2148.9	~2155.1	~2167.4	~530.2	~531.4	~533.9	~537.3	~540.7	
800 °C	S	~135.9	~136.9	~138.7	~146.5	~2148.5	~2155.1	~2167.5	~530.1	~531.4	~534.0	~537.3	~541.7
ES	~135.9	~136.8	~138.8	~146.5	~2148.7	~2154.9	~2167.4	~530.1	~531.2	~534.0	~536.6	~540.9	
NaPO_3_		~135.6	~136.5	~138.1	~146.4	~2149.0	~2155.1	~2167.3	~530.4	~531.4	~533.9	~537.3	~541.4
FePO_4_		~135.3	~136.6	~138.4	~145.8	–	–	–	–	–	–	~537.2	–
Fe_2_O_3_		–	–	–	–	–	–	–	~530.0	~531.5	–	~537.3	~541.5

Note: S = Surface; ES = Etched surface.

The error of peak position is no more than plus or minus 0.3 eV.

## References

[b1] Blau P. J. Elevated-temperature tribology of metallic materials. Tribol. Int. 43, 1023–1208 (2010).

[b2] Dohda K., Boher C., Rezai-Aria F. & Mahayotasanun N. Tribology in metal forming at elevated temperatures. Friction 3, 1–27 (2015).

[b3] Spikes H. The history and mechanisms of ZDDP. Tribol. Lett. 17, 469–489 (2004).

[b4] Martin J. M. Antiwear mechanism of zinc dithiophosphate: a chemical hardness approach. Tribol. Lett. 6, 1–8 (1999).

[b5] Gosvami N. N. . Mechanisms of antiwear tribofilm growth revealed *in situ* single-asperity sliding contacts. Science 348, 102–106 (2015). 25765069 10.1126/science.1258788

[b6] Andersson J., Larsson R., Almqvist A., Grahn M. & Minami I. Semi-deterministic chemo-mechanical model of boundary lubrication. Faraday Discuss. 156, 343–360 (2012). 23285638 10.1039/c2fd00132b

[b7] Willermet P. A., Dailey D. P. III, Carter R. O. & Zhu W. Mechanism of formation of antiwear films from zinc dialkyldithiophosphates. Tribol. Int. 28, 177–187 (1995).

[b8] Fulchiron R., Belyamani I., Otaigbe J. U. & Bounor-Legaré V. A simple method for tuning the glass transition process in inorganic phosphate glasses. Sci. Rep . 5, 8369 (2015). 25666949 10.1038/srep08369 PMC4322350

[b9] Warren O. L., Graham J. F., Norton P. R., Houston J. E. & Michalske T. A. Nanomechanical properties of films derived from zinc dialkyldithiophosphate. Tribol. Lett. 4, 189–198 (1998).

[b10] Tieu A. K., Wan S. H., Kong N., Zhu Q. & Zhu H. T. Excellent melt lubrication of alkali metal polyphosphate glass for high temperature applications. RSC Adv . 5, 1796–1800 (2015).

[b11] Tieu A. K. . Tribofilms generated from bulk polyphosphate glasses at elevated temperatures. Wear 330–331, 230–238 (2015).

[b12] Tieu A. K., Kong N., Wan S. H., Zhu Q. & Zhu H. T. The influence alkali metal polyphosphate on the tribological properties of heavily loaded steel on steel contacts at elevated temperatures. Adv. Mater. Interface 2, 1500032 (2015).

[b13] Ward W. C. & Denis R. A., inventors; The Lubrizol Corporation, assignee. Alkali metal borate and lubrication compositions thereof, United States patent US 8, 193, 130 B2, 2012 Jun 5.

[b14] Yatsuzuka Y., Murata M. &Tsuchiya T., inventors; Showa Shell Sekiyu K. K., assignee. Lubricant additive and lubricating grease composition containing the same, United States patent US 5, 877, 129, 1999 Mar 2.

[b15] Harrison J. J., Nelson K. D. & Buitrago J. A. Inventors; Chevron Oronite Company Llc, assignee. Dispersed hydrated potassium borate compositions having improved properties in lubricating oil compositions, United States patent US 6, 737, 387, 2004 May 18.

[b16] Yao J. B. & Dong J. X. Antioxidation synergism between alkali metal salts and arylamine compounds in synthetic lubricants. Tribol. T. 39, 498–500 (1996).

[b17] He Z. Y., Xiong L. P., Wang J. X., Qiu J. W. & Fu X. S. Tribological and oxidation properties study of a kind of sulfonate-modified nano Na_2_CO_3_ and K_2_CO_3_. Mater. Sci. Forum , 694, 98–102 (2011).

[b18] Yang J. H. & Wang X. B. K/Na-treated Fe-Cr-C hardfacing alloys with high-impact-abrasion resistance. Weld. J. 2, 103–107 (1995).

[b19] Martin J. M., Onodera T., Minfray C., Dassenoy F. & Mitamoto A. The origin of anti-wer chemistry of ZDDP. Faraday Discuss. 156, 311–323 (2012). 23285636 10.1039/c2fd00126h

[b20] Crobu M., Rossi A., Mangolini F. & Spencer N. D. Chain-length-identification strategy in zinc polyphosphate glasses by means of XPS and TOF-SIMS. Anal. Bioanal. Chem. 403, 1415–1432 (2012). 22451170 10.1007/s00216-012-5836-7

[b21] Minfray C. . Chemistry of ZDDP tribofilm by TOF-SIMS. Tribol. Lett. 17, 351–357 (2004).

[b22] Nicholls M. . The contribution of XANES spectroscopy to tribology. Can. J. Chem. 85, 816–830 (2007).

[b23] Najman M. N., Kasrai M. & Bancroft G. M. Chemistry of antiwear films from ashless thiophosphate oil additives. Tribolo. Lett. 17, 217–229 (2004).

[b24] Yin Z. F., Kasrai M., Bancroft G. M., Tan K. H. & Feng X. X-ray-absorption spectroscopic studies of sodium polyphosphate glasses. Phys. Rev. B 51, 742–750 (1995). 10.1103/physrevb.51.742 9978222

[b25] Pawlak Z. . External pressure in the hardening of phosphate in tribofilm on iron surfaces. JAMME 33, 35–40 (2009).

[b26] de Groot F. M. . Oxygen 1s x-ray-absorption edges of transition-metal oxides. Phys. Rev. B , 40, 5715–5723 (1989). 10.1103/physrevb.40.5715 9992609

[b27] Pollak M. . An *in-situ* study of the surfaces phase transitions of α-Fe_2_O_3_ by X-ray absorption spectroscopy at the oxygen K-edge. Nucl. Instr. Meth. Phys. Res. B 97, 383–386 (1995).

[b28] Wu Z. Y. . Characterization of iron oxides by x-ray absorption at the oxygen K edge using a full multiple-scattering approach. Phys. Rev. B 55, 2570–2577 (1997).

[b29] Magnien V. . Kinetics of iron oxidation in silicate melts: a preliminary XANES study. Chem. Geol. 213, 253–263 (2004).

[b30] Neuville D. R., Cormier L., Flank A.-M., Prado R. J. & Lagarde P. Na *K*-edge XANES spectra of minerals and glasses. Eur. J. Mineral . 16, 809–816 (2004).

[b31] Cibin G., Mottana A., Marcelli A. & Brigatti M. F. Potassium coordination in trioctahedral micas investigated by K-edge XANES spectroscopy. Mineral. Petrol. 85, 67–87 (2005).

[b32] Mathew V. . Amorphous iron phosphate: potential host for various charge carrier ions. NPG Asia Mater . 6, e138 (2014).

[b33] Minfray C. . Chemistry of ZDDP tribofilm by TOF-SIMS. Tribol. Lett. 17, 351–357 (2004).

[b34] Bingham P. A. & Barney E. R. Structure of iron phosphate glasses modified by alkali and alkaline earth additions: neutron and x-ray diffraction studies. J. Phys. Condens. Matter. 24, 175403 (2012). 22469777 10.1088/0953-8984/24/17/175403

[b35] Musinu A., Piccaluga G. &Pinna G. X-ray diffraction investigation of iron in sodium phosphate glasses. J. Phys. Chem. 100, 12462–12466 (1996).

[b36] Martin S. W. Review of the structures of phosphate glasses. Eur. J. Solid State Inorg. Chem. 28, 163–205 (1991).

[b37] Musinu A., Piccaluga G. & Pinna G. Structural properties of lead-iron phosphate glasses by X-ray diffraction. J. Non-Cryst. Solids 122, 52–68 (1990).

[b38] Shanjani Y., Hu Y., Pilliar R. M. & Toyserkani E. Mechanical characteristics of solid-freeform-fabricated porous calcium polyphosphate structures with oriented stacked layers. Acta Biomater. 7, 1788–1796 (2011). 21185409 10.1016/j.actbio.2010.12.017

[b39] Pereira G. . The role of the cation in antiwear films formed from ZDDP on 52100 steel. Tribol. Lett. 23, 109–119 (2006).

