# Corrigendum: Chemical nature of alkaline polyphosphate boundary film at heated rubbing surfaces

**DOI:** 10.1038/srep28214

**Published:** 2016-06-27

**Authors:** Shanhong Wan, A. Kiet Tieu, Qiang Zhu, Hongtao Zhu, Shaogang Cui, David R. G. Mitchell, Charlie Kong, Bruce Cowie, John A. Denman, Rong Liu

Scientific Reports 6: Article number: 26008; 10.1038/srep26008 published online: 05162015; updated: 06272016.

In this Article, Affiliation 6 is incorrectly listed as ‘Centre for Microscopy, Characterisation and Analysis, The University of Western Australia, Crawley, Australia’. The correct affiliation is listed below:

Centre for Microscopy, Characterisation and Analysis, Western Sydney University, Crawley, Australia.

