# Author Correction: Chemical nature of alkaline polyphosphate boundary film at heated rubbing surfaces

**DOI:** 10.1038/s41598-018-31338-0

**Published:** 2018-08-24

**Authors:** Shanhong Wan, A. Kiet Tieu, Qiang Zhu, Hongtao Zhu, Shaogang Cui, David R. G. Mitchell, Charlie Kong, Bruce Cowie, John A. Denman, Rong Liu

**Affiliations:** 1 Faculty of Engineering and Information Sciences, University of Wollongong, Wollongong, NSW 2522 Australia; 2 Electron Microscopy Centre, University of Wollongong, Wollongong, NSW 2522 Australia; 3 Electron Microscope Unit, The University of New South Wales, Sydney, NSW 2052 Australia; 4 Australian Synchrotron, 800 Blackburn Road, Clayton, Victoria 3168 Australia; 5 Future Industries Institute, University of South Australia, Mawson Lakes, SA 5095 Australia; 6 SIMS Facility, Office of the Deputy-Vice Chancellor (Research and Development), Western Sydney University, Locked Bag 1797, Penrith, NSW 2751 Australia

Correction to: *Scientific Reports* 10.1038/srep26008, published online 16 May 2016

In the original version of this Article, and in the Corrigendum published 27 June 2016, Rong Liu was incorrectly affiliated with ‘Centre for Microscopy, Characterisation and Analysis, The University of Western Australia, Crawley, Australia’ and ‘Centre for Microscopy, Characterisation and Analysis, Western Sydney University, Crawley, Australia’ respectively. The correct affiliation is listed below.

SIMS Facility, Office of the Deputy-Vice Chancellor (Research and Development), Western Sydney University, Locked Bag 1797, Penrith, NSW, 2751, Australia.

This error has now been corrected in the PDF and HTML versions of the Article.

